# Birth Prevalence of Sickle Cell Disease and County-Level Social Vulnerability — Sickle Cell Data Collection Program, 11 States, 2016–2020

**DOI:** 10.15585/mmwr.mm7312a1

**Published:** 2024-03-28

**Authors:** Mariam Kayle, Audrey L. Blewer, Wei Pan, Jennifer A. Rothman, Carri S. Polick, Joshua Rivenbark, Elliott Fisher, Camila Reyes, John J. Strouse, Shelby Weeks, Jay R. Desai, Angela B. Snyder, Mei Zhou, Ankit Sutaria, Jhaqueline Valle, Sophia S. Horiuchi, Marci K. Sontag, Joshua I. Miller, Ashima Singh, Mahua Dasgupta, Isaac A. Janson, Najibah Galadanci, Sarah L. Reeves, Krista Latta, Isabel Hurden, Shamaree J. Cromartie, Allison P. Plaxco, Ayesha Mukhopadhyay, Matthew P. Smeltzer, Mary Hulihan

**Affiliations:** ^1^Duke University School of Nursing, Durham, North Carolina; ^2^Duke University School of Medicine, Durham, North Carolina; ^3^Durham VA Health Care System, Durham, North Carolina; ^4^Division of Hematology, University of North Carolina, Chapel Hill, North Carolina; ^5^Duke Office of Clinical Research, Duke University School of Medicine, Durham, North Carolina; ^6^Division of Public Health, North Carolina Department of Health and Human Services; ^7^Minnesota Department of Health; ^8^Georgia Health Policy Center, Georgia State University, Atlanta, Georgia; ^9^Georgia Department of Public Health; ^10^Tracking California, Public Health Institute, Richmond, California; ^11^Center for Public Health Innovation at CI International, Littleton, Colorado; ^12^Medical College of Wisconsin, Milwaukee, Wisconsin; ^13^Indiana Hemophilia and Thrombosis Center, Indianapolis, Indiana; ^14^University of Alabama at Birmingham, Birmingham, Alabama; ^15^Susan B. Meister Child Health Evaluation and Research Center, Department of Pediatrics, University of Michigan, Ann Arbor, Michigan; ^16^Michigan Department of Health and Human Services; ^17^Virginia Department of Health; ^18^Division of Epidemiology, Biostatistics and Environmental Health, School of Public Health, The University of Memphis, Memphis, Tennessee; ^19^Division of Blood Disorders and Public Health Genomics, National Center on Birth Defects and Developmental Disabilities, CDC.

**Summary**

**What is already known about the topic?**

Approximately one in every 365 Black or African American (Black) newborns in the United States has sickle cell disease (SCD), a condition associated with complex health needs.

**What is added by this report?**

During 2016–2020, 3,305 cases of SCD among newborns were recorded across 11 states participating in the Sickle Cell Data Collection program (SCD birth prevalence = 28.54 per 10,000 [one in every 350] non-Hispanic Black newborns). Approximately two thirds of mothers of newborns with SCD resided in counties with high or very high social vulnerability.

**What are the implications for public health?**

Implementation of tailored interventions, including increasing access to transportation, improving housing, and advancing equity in high vulnerability areas, could facilitate care and improve health outcomes for children with SCD.

## Abstract

Sickle cell disease (SCD) remains a public health priority in the United States because of its association with complex health needs, reduced life expectancy, lifelong disabilities, and high cost of care. A cross-sectional analysis was conducted to calculate the crude and race-specific birth prevalence for SCD using state newborn screening program records during 2016–2020 from 11 Sickle Cell Data Collection program states. The percentage distribution of birth mother residence within Social Vulnerability Index quartiles was derived. Among 3,305 newborns with confirmed SCD (including 57% with homozygous hemoglobin S or sickle β-null thalassemia across 11 states, 90% of whom were Black or African American [Black], and 4% of whom were Hispanic or Latino), the crude SCD birth prevalence was 4.83 per 10,000 (one in every 2,070) live births and 28.54 per 10,000 (one in every 350) non-Hispanic Black newborns. Approximately two thirds (67%) of mothers of newborns with SCD lived in counties with high or very high levels of social vulnerability; most mothers lived in counties with high or very high levels of vulnerability for racial and ethnic minority status (89%) and housing type and transportation (64%) themes. These findings can guide public health, health care systems, and community program planning and implementation that address social determinants of health for infants with SCD. Implementation of tailored interventions, including increasing access to transportation, improving housing, and advancing equity in high vulnerability areas, could facilitate care and improve health outcomes for children with SCD.

## Introduction

Sickle cell disease (SCD) is an inherited blood disorder caused by mutations in the hemoglobin subunit beta (*HBB*) gene and is associated with premature mortality (*1*) and significant morbidity, including vasoocclusive pain, stroke, and multiorgan damage. The protective association between variants in *HBB* and severe *Plasmodium falciparum* malaria results in a higher prevalence of *HBB* mutations in geographic areas with high malaria prevalences (*2*). The combination of this protective effect and the historical trans-Atlantic slave trade has resulted in SCD primarily affecting Black or African American (Black) persons in the United States, leading to exacerbation of high disease-associated morbidity in groups affected by structural racism and health inequities. Social determinants of health further contribute to poor outcomes among persons with SCD (*3*,*4*). Because of the associated health inequities, high risk of lifelong disabilities, and high cost of care, managing SCD remains a major national public health priority (*5*).

Although universal newborn screening for hemoglobinopathies, including SCD, has been implemented nationally since 2006 (*6*), SCD birth prevalence data remain scarce. The most recent race-specific birth prevalence (one in 365 Black newborns) is based on 2007 data (*7*). Data from 2015–2017 indicate an overall SCD birth prevalence of one in 2,024 U.S. newborns (*8*). The CDC-funded Sickle Cell Data Collection (SCDC) program is well suited to estimate SCD birth prevalence. At the time of this analysis, SCDC included 11 state-level surveillance programs from the southern, midwestern, and western United States. The programs collect and analyze data on newborns with SCD, including SCD type and geographic location, allowing for the assessment of county-level socioeconomic conditions that influence health outcomes among infants with SCD. Identifying these conditions can guide the planning and implementation of public health, health care systems, and community programs supporting persons with SCD. This report analyzed state newborn screening program records from 2016–2020 from 11 SCDC program states to provide updated crude and race-specific SCD prevalence among newborns and to describe the percentage of newborns with SCD by county-level socioeconomic characteristics among these states.

## Methods

### Data Sources

State newborn screening records from 2016–2020, combined with birth certificate data and confirmation testing results when available, were used to identify the most recent data across 11 SCDC program states (Alabama, California, Colorado, Georgia, Indiana, Michigan, Minnesota, North Carolina, Tennessee, Virginia, and Wisconsin). Newborns with a confirmed diagnosis of SCD were included if the infant’s birth and their mother’s residence county were within the SCDC program state. Confirmed SCD diagnosis was based on a positive Clinical Laboratory Improvement Amendments (CLIA)–certified laboratory SCD test result reported by a state newborn screening program with confirmatory testing or clinical diagnosis by a physician and documented confirmatory CLIA-certified laboratory testing after the newborn period. The mother’s residence county and infant’s race, ethnicity, sex, date of birth, and SCD type were extracted from newborn screening records or birth certificate data. Infants’ race, ethnicity, and SCD type were based upon state-specific methodologies across newborn screening programs (*9*). The total number of live births and of live births among non-Hispanic Black persons by county were obtained from each state’s health department.

### Data Analysis

Crude SCD birth prevalence (calculated by dividing the number of newborns with SCD by the total number of live births) and SCD birth prevalence among non-Hispanic Black newborns (calculated by dividing the number of Black* newborns with SCD by the total number of live births among non-Hispanic Black^†^ persons) were reported per 10,000 newborns. Prevalence rates were calculated overall and by state.

County Social Vulnerability Index (SVI) characteristics were quantified using the 2020 state-specific SVI databases based on the birth mother’s county of residence at birth. The state-specific SVI ranks each county relative to other counties within the state on 16 social factors. Percentile ranking values range from 0 to 1, with higher values indicating greater vulnerability. An overall SVI percentile ranking as well as percentile rankings measured on four themes (socioeconomic status, household characteristics, racial and ethnic minority status, and housing type and transportation)^§^ were categorized into quartiles from least to most vulnerable: low (0 to 0.25), medium (>0.25 to 0.5), high (>0.5 to 0.75), and very high (>0.75 to 1.0) vulnerability. The percentage distribution of birth mother residence within SVI quartiles was derived for the overall SVI measure and by SVI themes.

SAS (version 9.4; SAS Institute) was used for all analyses. Institutional review boards or ethics committees overseeing each state program either determined the analysis to be outside purview as public health surveillance or exempt and approved a waiver of consent. This activity was reviewed by CDC, deemed not research, and was conducted consistent with applicable federal law and CDC policy.^¶^

## Results

### Demographics and Birth Prevalence

During 2016–2020, a total of 3,305 SCD-affected newborns were recorded across the 11 SCDC program states (Table 1). The highest number of SCD-affected newborns (758) occurred in Georgia, followed by North Carolina (435), California (419), and Alabama (386). Approximately 50% of newborns with SCD were male, 90% were Black, and 4% were Hispanic or Latino. Overall, 1,882 (57%) infants had homozygous hemoglobin S (HbSS) or sickle β-null thalassemia (HbSβ^0^), 28% had hemoglobin SC disease, and 10% had sickle β-plus thalassemia (HbSβ^+^) or another SCD type.** Across the 11 states, crude SCD birth prevalence was 4.83 per 10,000 (one in every 2,070) live births (Table 2). SCD birth prevalence among non-Hispanic Black newborns was 28.54 per 10,000 (one in every 350) live births.

**TABLE 1:** Number of newborns with sickle cell disease, by sex, race, ethnicity, and confirmed sickle cell disease type (N = 3,305) — 11 Sickle Cell Data Collection program states, 2016–2020

State	Total no. of newborns with SCD	Sex			Race			Ethnicity*			Confirmed SCD type			
		Female	Male	Unk	Black or African American	Other	Unk	Hispanic or Latino	NH	Unk	HbSS or HbSβ^0^	HbSC	HbSβ^+^ or other	Unk
Alabama	**386**	179	159	—^†^	312	—	—	—	52	—	212	85	30	59
California	**419**	207	212	—	374	—	—	—	371	—	219	133	67	0
Colorado	**66**	27	39	—	54	—	—	—	56	—	40	18	—	—
Georgia	**758**	398	360	—	711	—	—	—	667	—	409	207	53	89
Indiana	**175**	74	101	—	144	—	—	—	142	—	108	51	16	0
Michigan	**315**	160	155	—	284	—	—	—	250	—	180	86	49	0
Minnesota	**90**	46	44	—	84	—	—	—	85	—	54	15	—	—
North Carolina	**435**	196	239	—	395	—	—	—	412	—	268	129	19	19
Tennessee	**224**	113	111	—	215	—	—	—	212	—	136	63	25	0
Virginia	**321**	161	159	—	289	—	—	—	218	—	188	106	27	0
Wisconsin	**116**	56	60	—	108	—	—	—	104	—	68	28	—	—
**Total (row %)**	**3,305** **(100)**	**1,617** **(48.9)**	**1,639** **(49.6)**	**49** **(1.5)**	**2,970** **(89.9)**	**198** **(6.0)**	**137** **(4.1)**	**134** **(4.1)**	**2,569** **(77.7)**	**602** **(18.2)**	**1,882** **(56.9)**	**921** **(27.9)**	**317** **(9.6)**	**185** **(5.6)**

**Abbreviations:** HbSC = hemoglobin SC disease; HBSS = sickle cell homozygous hemoglobin S; HbSβ^0^ = sickle beta-null thalassemia; HbSβ^+^ = sickle beta-plus thalassemia; NH = non-Hispanic; SCD = sickle cell disease; Unk = unknown.

* Ethnicity was unknown in 18% of cases; therefore, ethnicity results should be interpreted with caution.

^†^ Dashes indicate data was censored because counts were <11 in one or more cells.

**TABLE 2:** Sickle cell disease birth prevalence overall and among non-Hispanic Black or African American infants — 11 Sickle Cell Data Collection program states, 2016–2020

State	Total no. of newborns with SCD	Total no. of live births	Overall crude prevalence* of newborns with SCD	No. of Black newborns with SCD^†^	No. of NH Black live births^§^	Prevalence^¶^ of NH Black newborns with SCD
Alabama	**386**	**292,038**	13.22	312	91,467	34.11
California	**419**	**2,281,910**	1.84	374	136,485	27.40
Colorado	**66**	**540,370**	1.22	54	17,541	30.79
Georgia	**758**	**633,778**	11.96	711	228,199	31.16
Indiana	**175**	**406,382**	4.31	144	52,827	27.26
Michigan	**315**	**547,020**	5.76	284	107,109	26.52
Minnesota	**90**	**335,154**	2.69	84	43,514	19.30
North Carolina	**435**	**595,301**	7.31	395	143,595	27.51
Tennessee	**224**	**401,622**	5.58	215	81,995	26.22
Virginia	**321**	**493,627**	6.50	289	104,973	27.53
Wisconsin	**116**	**319,625**	3.63	108	33,073	32.66
**Total**	**3,305**	**6,846,827**	**4.83**	**2,970**	**1,040,778**	**28.54**

**Abbreviations**: Black = Black or African American; NH = non-Hispanic; SCD = sickle cell disease; SCDC = Sickle Cell Disease Collection.

* Crude prevalence of newborns with SCD were calculated by dividing the number of newborns with SCD by number of live newborns in each state and multiplying by 10,000.

^†^ The number of newborns with SCD was reported to SCDC programs with separate variables for race and ethnicity. As a result, the numerator in the calculation of SCD birth prevalence includes all Black newborns with SCD, regardless of ethnicity.

^§^ The number of live births was reported to SCDC programs with a single, combined variable for race and ethnicity. As a result, the denominator in the calculation of SCD birth prevalence includes only NH Black newborns.

^¶^ Prevalence of NH Black newborns with SCD was calculated by dividing the number of Black newborns with SCD by number of live births among NH Black persons in each state during 2016–2020 and multiplying by 10,000. Birth rates among NH Black persons for Alabama, Colorado, and Indiana should be interpreted with caution because race was unknown for 9%–14% of newborns with SCD in these states; thus, the NH Black SCD birth prevalence for these states could be underestimated.

### Social Vulnerability

Among all mothers of newborns with SCD, approximately two thirds (67%) lived in counties with high or very high social vulnerability (Figure). In five of the 11 SCDC program states, more than one half of birth mothers resided in very high SVI counties (Wisconsin [86%], Indiana [82%], Michigan [61%], Tennessee [58%], and California [56%]) (Supplementary Figure, https://stacks.cdc.gov/view/cdc/151052). Approximately one half (49%) of mothers of newborns with SCD resided in areas with high or very high socioeconomic vulnerability and household characteristic vulnerability (56%). In addition, approximately two thirds (64%) of mothers resided in counties with high or very high housing type and transportation vulnerability, and 89% resided in counties with high or very high racial and ethnic minority status vulnerability (Figure).

**FIGURE:**
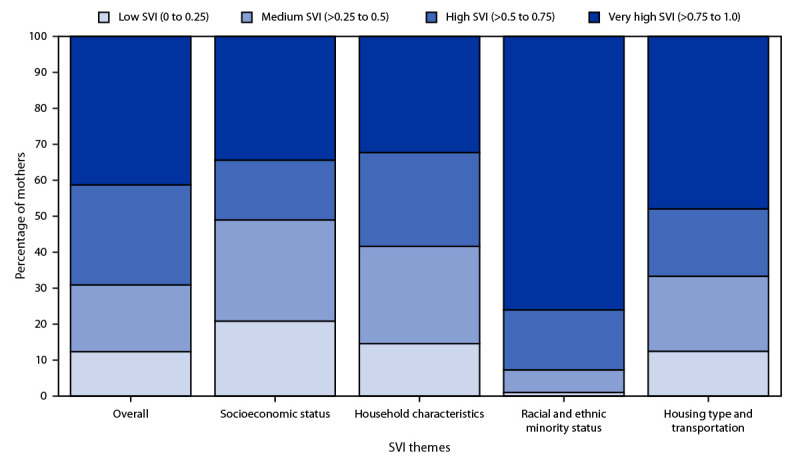
Percentage of mothers of newborns with sickle cell disease (N = 3,305), by overall and theme-specific Social Vulnerability Index quartiles* — 11 Sickle Cell Data Collection program states,^†^ 2016–2020 **Abbreviation**: SVI = Social Vulnerability Index. * The 2020 state-ranked SVI datasets based on mother’s county of residence at birth were used (https://www.atsdr.cdc.gov/placeandhealth/svi/data_documentation_download.html). SVI values were state-specific and ranked each county relative to other counties within a state. The SVI ranks counties based on 16 social factors. Percentile ranking values range from 0 to 1, with higher values indicating greater vulnerability. Themes were socioeconomic status (<150% of the federal poverty level, unemployed, housing cost burden, no high school diploma, and no health insurance), household characteristics (age ≥65 years, age ≤17 years, civilian with a disability, single-parent households, and English language proficiency), racial and ethnic minority status (non-Hispanic American Indian or Alaska Native, non-Hispanic Asian, non-Hispanic Black or African American, non-Hispanic Native Hawaiian or other Pacific Islander, non-Hispanic two or more races, Hispanic or Latino of any race, and non-Hispanic other races), and housing type and transportation (multiunit structures, mobile homes, crowding, no vehicle, and group quarters). For 127 (4%) mothers of newborns with sickle cell disease, the county of residence at birth was unknown. ^†^ Alabama, California, Colorado, Georgia, Indiana, Michigan, Minnesota, North Carolina, Tennessee, Virginia, and Wisconsin.

## Discussion

In this analysis of birth prevalence and social vulnerability ranking of newborns with SCD across 11 SCDC program states, SCD affected one in every 2,070 newborns overall and one in every 350 non-Hispanic Black newborns. These findings align with previously reported estimates of SCD affecting one in every 2,024 newborns overall and one in every 365 non-Hispanic Black newborns (*7*,*8*); however, they expand on those reports by using more recent data from 2016–2020.

The finding that most mothers lived in counties with high or very high SVI highlights the insights that county-level data can provide to public health policymakers when considering the support that community-based programs can deliver to meet the complex health needs of newborns with SCD and their caregivers. The majority (64%) of mothers of newborns with SCD resided in counties with high or very high housing type and transportation social vulnerability, underscoring potential strategies to serve these communities, including medical transportation programs or development of innovative care models to facilitate access to comprehensive SCD care. For example, improving flexibility in scheduling of medical transportation, providing reimbursement for use of existing public transportation such as rideshares, and partnering with local faith- and community-based organizations for medical transport have the potential to improve access to care. Moreover, understanding the geographic location of SCD-affected newborns within a state can help guide specialty and primary care efforts to improve access to SCD care. Together, these findings provide data to Medicaid programs, the primary payer for SCD care,^††^ as they collaborate with state agencies to consider the effect of housing and transportation vulnerabilities on infants with SCD. These Medicaid programs can also support partnerships created by public health programs, the communities they serve, and community-based organizations to ascertain specific resource needs of SCD-affected populations, as well as where and how resources can be deployed to drive more equitable outcomes.

### Limitations

The findings in this report are subject to at least four limitations. First, because of the time needed to ascertain SCD type by public health surveillance systems, state newborn screening data are subject to a 3-year time lag. Despite this lag, SCD counts among newborns did not fluctuate significantly between years. Second, missing data hampered the ability to further disaggregate race and ethnicity categories, which might be important to understanding differential birth prevalences across states and tailoring programs to different racial and ethnic communities. Third, SVI was examined at the county level as opposed to U.S. Census Bureau tract level, which could mask variations of SVI within counties. Finally, SVI metrics are at the county level rather than the person level and might not reflect a comprehensive assessment of specific services needed by infants with SCD.

### Implications for Public Health Practice

Understanding characteristics of the geographic residence of infants with SCD and their caregivers is critical to guiding local and state health departments and health agencies in prioritizing and developing programs that can mitigate specific social determinants of health and their attendant inequities. Specifically, these data highlight the potential need to consider tailored interventions in high vulnerability areas to increase access to transportation, improve housing, and advance equity for infants with SCD. Developing programs in partnership with communities and community-based organizations is critical to allocating needed resources and determining how they might be effective in improving health outcomes for children with SCD.

## Footnotes

* The number of newborns with SCD was reported to the SCDC programs with separate variables for race and ethnicity. As a result, the numerator in the calculation of SCD birth prevalence includes all Black newborns with SCD, regardless of ethnicity.

† The number of live births was reported to the SCDC programs with a single, combined variable for race and ethnicity. As a result, the denominator in the calculation of SCD birth prevalence includes only non-Hispanic Black newborns. Because the count of Black newborns with SCD included all ethnicities but the count of all Black live births included non-Hispanic newborns only, the non-Hispanic Black SCD birth prevalence could be overestimated.

§
https://www.atsdr.cdc.gov/placeandhealth/svi/data_documentation_download.html

¶ 45 C.F.R. part 46.102(l)(2), 21 C.F.R. part 56; 42 U.S.C. Sect. 241(d); 5 U.S.C. Sect. 552a; 44 U.S.C. Sect. 3501 et seq.

** Hemoglobin SS disease (HbSS) is usually the most severe type of SCD; children inherit a hemoblobin S gene from each parent. Persons with HbSC disease inherit a hemoglobin S gene from one parent, and a gene for abnormal hemoglobin C from the other parent; HbSC is usually milder than HbSS. Children with HbSβ inherit the hemoglobin S gene from one parent and a β thalassemia gene from the other parent. HbSβ^0^ is usually more severe than is HbSβ^+^. Rarer SCD types include HbSD, HbSE, and HbSO, in which an abnormal D, E, or O hemoglobin gene is inherited from one parent, and the hemoglobin S gene is inherited from the other parent. https://www.cdc.gov/ncbddd/sicklecell/facts.html

††
https://www.ncbi.nlm.nih.gov/books/NBK547764/
